# Case series: TRAP sequence

**DOI:** 10.4103/0971-3026.45352

**Published:** 2009-02

**Authors:** M. Chandramouly, Namitha

**Affiliations:** Department of Radiodiagnosis, RAGAVS Diagnostic and Research Centre Pvt Ltd., Sadguru Complex, No.14, 27th Cross, 4th Block West, Jayanagar, Bangalore - 560 011, India

**Keywords:** Donor and recipient twin, TRAP sequence, twin pregnancy, ultrasound

## Abstract

TRAP (twin reversed arterial perfusion) sequence / acardiac twinning is a rare anomaly that occurs in monozygotic monochorionic twins with an incidence of 1% and in 1 in 35,000 pregnancies overall. The anomalous twin appears as a heterogeneous mass, simulating a teratoma or intrauterine fetal demise. As the normal twin faces increased morbidity and mortality, antenatal diagnosis with gray-scale examination, and Doppler confirmation of the diagnosis of TRAP sequence in twin pregnancies, aids in proper prenatal management. We report two cases of TRAP sequence that we encountered over a single month, with the two cases having different outcomes.

## Introduction

TRAP (twin reversed arterial perfusion) sequence / acardiac twinning is a rare anomaly that occurs in monozygotic monochorionic twins with an incidence of 1% and in 1 in 35,000 pregnancies overall.[1] In the TRAP sequence, the normal twin donates blood to the abnormal twin through its umbilical arteries via vascular anastomoses in the placenta. The anomalous twin appears as a heterogeneous mass, simulating a teratoma or even intrauterine fetal demise. Diagnosis is necessary for proper prenatal management and can be established by Doppler examination of the umbilical artery of the abnormal twin. We report two cases of TRAP sequence that we encountered over a single month, with the two cases having different outcomes.

## Case Reports

### Case 1

**Figure 1:**
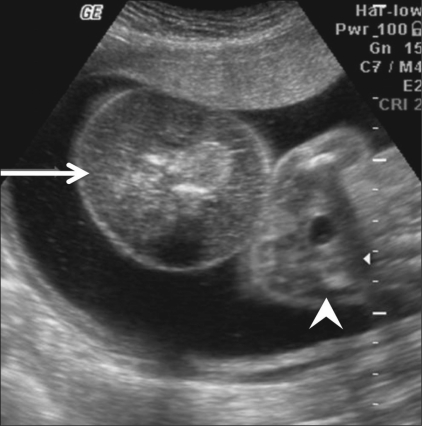
Case 1: Transabdominal USG shows a monochorionic monoamniotic twin gestation with a normal fetus (arrow) and the circular abdominal stump of the abnormal fetus demonstrating heterogeneous echotexture, without any discernable internal organs (arrowhead)

**Figure 2 (A, B):**
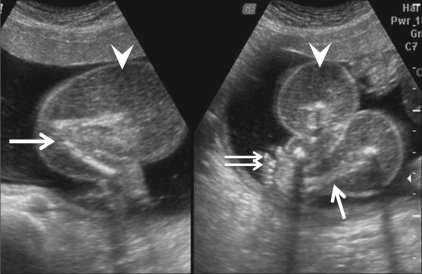
Case 1: Longitudinal (A) and oblique axial (B) scans show well-formed lower limbs (arrows) and a foot (double arrow) of the abnormal fetus with diffuse massive soft tissue edema (arrowheads)

**Figure 3:**
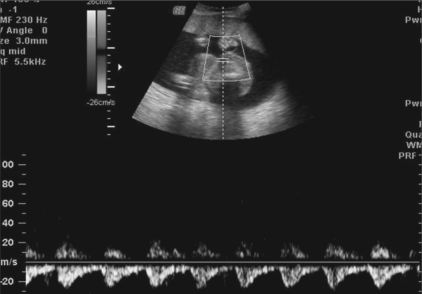
Case 1: Pulsed Doppler of the umbilical artery of the abnormal fetus shows reversal of flow on the spectral waveform

A 29-year-old lady, G_4_P_0_L_0_A_3_, came for a second trimester anomaly scan. USG revealed a monochorionic, monoamniotic twin gestation with one normal appearing fetus of gestational age 22–23 weeks [Figure 1]. The patient also had polyhydramnios and a single large placental mass located in the fundo-anterior region [Figure 1]. The second fetus had an incompletely formed skeleton, with no head, upper limbs, heart, or thoracic structures. It had an abdominal stump without any intraabdominal organs; there were two well-developed lower limbs which showed massive, diffuse, soft tissue edema [Figure 2]. On color Doppler imaging, the umbilical artery in the abnormal fetus showed reversal of flow on the spectral graph [Figure 3]. The umbilical cord of the abnormal fetus had only a single umbilical artery. This appearance was typical of a TRAP sequence with an acardiac parabiotic twin (acardius acephalus / acardius chorioangiopagus parasiticus). This patient went into premature labor secondary to polyhydramnios at 27 weeks of gestation and delivered an amorphous mass and a normal-appearing fetus which, however, died soon thereafter.

### Case 2

**Figure 4:**
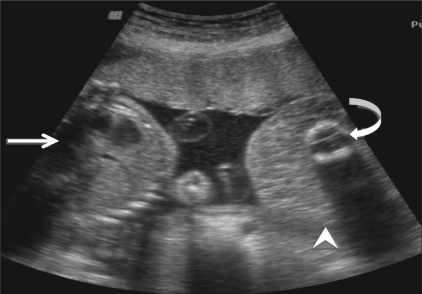
Case 2: Transabdominal USG shows a monochorionic, monoamniotic twin gestation with a normal fetus (arrow), an abnormal fetus [seen as an isoechoic mass without any internal organs (arrowheads)], and an echogenic structure casting a distal acoustic shadow, possibly representing the spine of the abnormal fetus (curved arrow)

**Figure 5 (A–D):**
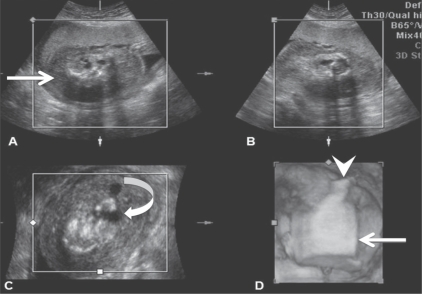
Case 2: Three orthogonal planes [i.e., longitudinal (A), transverse (B), coronal (C)] and the reconstructed 3D (D) images of the abnormal fetus show a blind-ending abdominal stump (arrow) appearing as a mass, with a small, single lower limb (arrowhead) and a cystic structure—possibly the fetal bladder (curved arrow)

**Figure 6:**
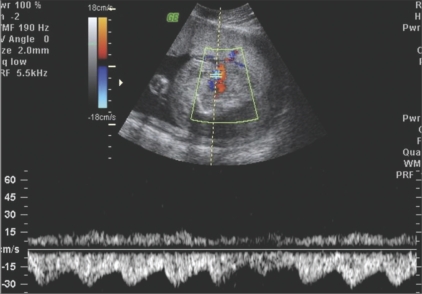
Case 2: Pulsed Doppler of the umbilical artery of the abnormal fetus shows reversalof flow on the spectral waveform

A 26-year-old lady, a primigravida, was referred for a routine second trimester anomaly scan. USG revealed a monochorionic, monoamniotic twin gestation with one normal appearing fetus of gestational age 23–24 weeks [Figure 4]. A single large placental mass was seen in the fundo-anterior region [Figure 4]. The second fetus was an amorphous heterogeneous mass; it had an abdominal stump with a cystic structure, possibly the urinary bladder, and an echogenic structure that resembled the spine [Figure 4 and 5]. It had a single small lower limb [Figure 5]. On pulsed Doppler imaging, the single umbilical artery in the abnormal fetus showed reversal of flow on the spectral graph [Figure 6]. This appearance was compatible with the diagnosis of a TRAP sequence with an acardiac parabiotic twin. This patient was followed-up with serial USG examinations and she had a successful outcome, with the delivery of a normal fetus and an amorphous mass at term.

## Discussion

In the TRAP sequence, the normal twin ‘pumps’ or ‘donates’ blood to the abnormal twin, which is called the ‘recipient’ or ‘perfused’ twin through abnormal artery-to-artery or venous-to-venous communications in the placenta.[2] There is a reversal of flow in the recipient twin, with relatively oxygenated blood flowing from the abnormal anastomosis to the umbilical artery; the flow then proceeds cranially, leaving the fetus via the umbilical vein; hence the term, TRAP sequence. This finding can be confirmed by pulsed Doppler of the umbilical artery of the recipient twin, which will reveal reversal of flow on the spectral waveform [Figures 3 and 6]. In 75% of cases, the umbilical cord of the recipient twin contains a single umbilical artery [Figure 6].[3]

As a result of imbalance of the interfetal circulation, the caudal aspect of the perfused fetus receives blood with relatively more nutrients and oxygen than the upper torso, resulting in better development of the pelvis and lower extremities in the acardiac fetus. Fully desaturated blood then flows in a retrograde fashion to the upper body and head, leading to maldevelopment of the heart, head, and upper torso, which are either completely absent or severely deficient.[2] Therefore, on USG it appears as a heterogeneous mass, simulating a teratoma or intrauterine fetal demise.[4] Chromosomal anomalies may be present in up to 50% of cases of acardiac fetus.[1] The acardiac twin usually has a dorsal cystic hygroma but in our series this was not present.[5]

Classification of acardiac twinning is as follows:

- Hemiacardius – if the heart is incompletely formed
- Holoacardius – if the heart is absent
- Another type of classification is as follows:
- Acardius anceps – when head is poorly formed
- Acardius acephalus – if the head is absent
- Acardius acormus – presence of head only
- Acardius amorphous – unrecognizable amorphous mass[3]

Congenital anomalies are present in about 9% of pump twins.[1] The overall perinatal mortality of pump twins is 50-55%, being usually due to either polyhydramnios leading to premature delivery or secondary to congestive cardiac failure[2]; high-output cardiac failure develops due to the increased cardiac output secondary to the abnormal interfetal circulation. This high cardiac output also increases perfusion of the fetal kidneys, resulting in overproduction of fetal urine and polyhydramnios. When the ratio of the weight of the acardiac fetus to the weight of the donor fetus is greater than 70%, the incidence of preterm delivery is 90%, that of polyhydramnios is 40%, and that of congestive heart failure in the pump twin is 30%; in comparison, the corresponding rates are 75, 30, and 10%, respectively, when the ratio is less than 70%.[1] The weight of the acardiac twin cannot be calculated from the values of the head circumference, abdominal circumference, and femur length using the standard formulae (such as Hadlock's); instead, the weight (in grams) of the acardiac twin is calculated with the following formula: (1.2 × longest length^2^) - (1.7 × longest length).[5] One prognostic factor is the resistive index (RI) of the umbilical arteries. If the difference between the pump twin and the acardius is more than 0.20, the prognosis is good.[5,6]

Prenatal treatment involves occlusion of blood flow to the acardiac twin by endoscopic (fetoscopic) ligation or laser coagulation of the umbilical cord, bipolar cord cauterization, or intrafetal radiofrequency ablation.[3] The indications for prenatal treatment include polyhydramnios, cardiac dysfunction, hydrops of the pump twin, or a relatively large weight of the acardiac twin.[5]
